# Treating periprocedural bleeding in patients with cirrhosis

**DOI:** 10.1007/s11239-023-02941-4

**Published:** 2024-01-28

**Authors:** Antoni Sabate, Ecaterina Scarlatescu

**Affiliations:** 1 Anesthesia Department, Bellvitge University Hospital, University of Barcelona, Idibell, Barcelona, Spain; 2 Anesthesia and Intensive Care Department, University of Medicine and Pharmacy Carol Davila, Bucharest, Romania; 3 Department of Anesthesia and Intensive Care Medicine, Fundeni Clinical Institute, Bucharest, Romania

**Keywords:** Periprocedural bleeding, Cirrhosis, Coagulation, Liver transplant, Viscoelastic testing

## Abstract

Patients with cirrhosis are known to have an abnormal coagulation status, which is a particular concern when planning invasive procedures in which blood loss is possible or predictable. Careful consideration must be given to the bleeding risk for each individual patient and coagulation management strategies should be established in advance of procedural interventions, where possible. Perioperative clinical decision-making should utilize viscoelastic testing in addition to usual assessments, where possible, and focus on the well-established three pillars of patient blood management: optimization of erythropoiesis, minimization of bleeding and blood loss, and management of anemia. Restrictive transfusion policies, careful hemostatic monitoring, and a proactive approach to predicting and preventing bleeding on an individual patient basis should be central to managing perioperative bleeding in the fragile patient population with cirrhosis. This review discusses coagulation assessments and bleeding management techniques necessary before, during, and after surgical interventions in patients with cirrhosis, and provides expert clinical opinion and physician experience on the perioperative management of these patients.

## Keypoints

Periprocedural bleeding is a potential complication in patients with cirrhosis.

Viscoelastic testing (VET) may help to assess coagulopathy and bleeding risk.

Perioperative infusion strategies should generally be restrictive, but re-assessed on an individual basis.

In future, publication of specific anemia and VET-guided bleeding management clinical studies of large populations, consensus statements and guidelines would be beneficial.

## Introduction

The liver synthesizes coagulation factors and fibrinolytic proteins crucial for hemostasis. However, patients with advanced liver disease have rebalanced hemostasis due to net changes in both pro- and anticoagulant pathways providing a delicate equilibrium, which can be easily disturbed leading to bleeding and thrombotic complications depending on clinical events [1]. Furthermore, liver diseases can have varying effects on hemostasis, with mild to moderate thrombocytopenia often an indicator of severe liver disease. Other common findings in patients with cirrhosis include decreased levels of fibrinolytic proteins, reduced levels of coagulation factors and anticoagulant proteins produced in the liver, elevated factor VIII and von Willebrand factor levels, and decreased ADAMTS-13 (A Disintegrin And Metalloprotease with ThromboSpondin-1 domain) levels [1].

Standard coagulation tests (SCTs) have traditionally been used for diagnostic and management purposes in patients with cirrhosis. However, viscoelastic testing (VET) appears superior to SCTs in assessing cirrhotic coagulopathy, better reflecting the rebalanced hemostatic status of cirrhotic patients and improving the accuracy of coagulation test results. Classic SCTs measurements such as prothrombin time, international normalized ratio (INR), and activated partial thromboplastin time can provide a misleading indication of bleeding tendency and are not effective predictors of future bleeding events in patients with cirrhosis [1, 2].

VET on whole-blood samples is increasingly being used to support coagulation management in patients with liver disease [1]. In addition, VET provides a more accurate overall insight, in real-time, into the overall coagulation status than SCT allowing a decreased use of blood products in cirrhotic patients at risk of or experiencing bleeding, without increasing complications [2]. There are discrepancies between SCT and VET results, particularly in the case of patients with cirrhosis whereby SCTs show hypocoagulability, whereas VET indicates normal or even increased clot formation, which would counteractively suggest that prophylactic prevention of bleeding may not be necessary [1, 2]. Diagnostic and point-of-care testing, including VET, help clinicians to make decisions on treatment therapies before, during, and after bleeding events occur.

**Fig. 1:**
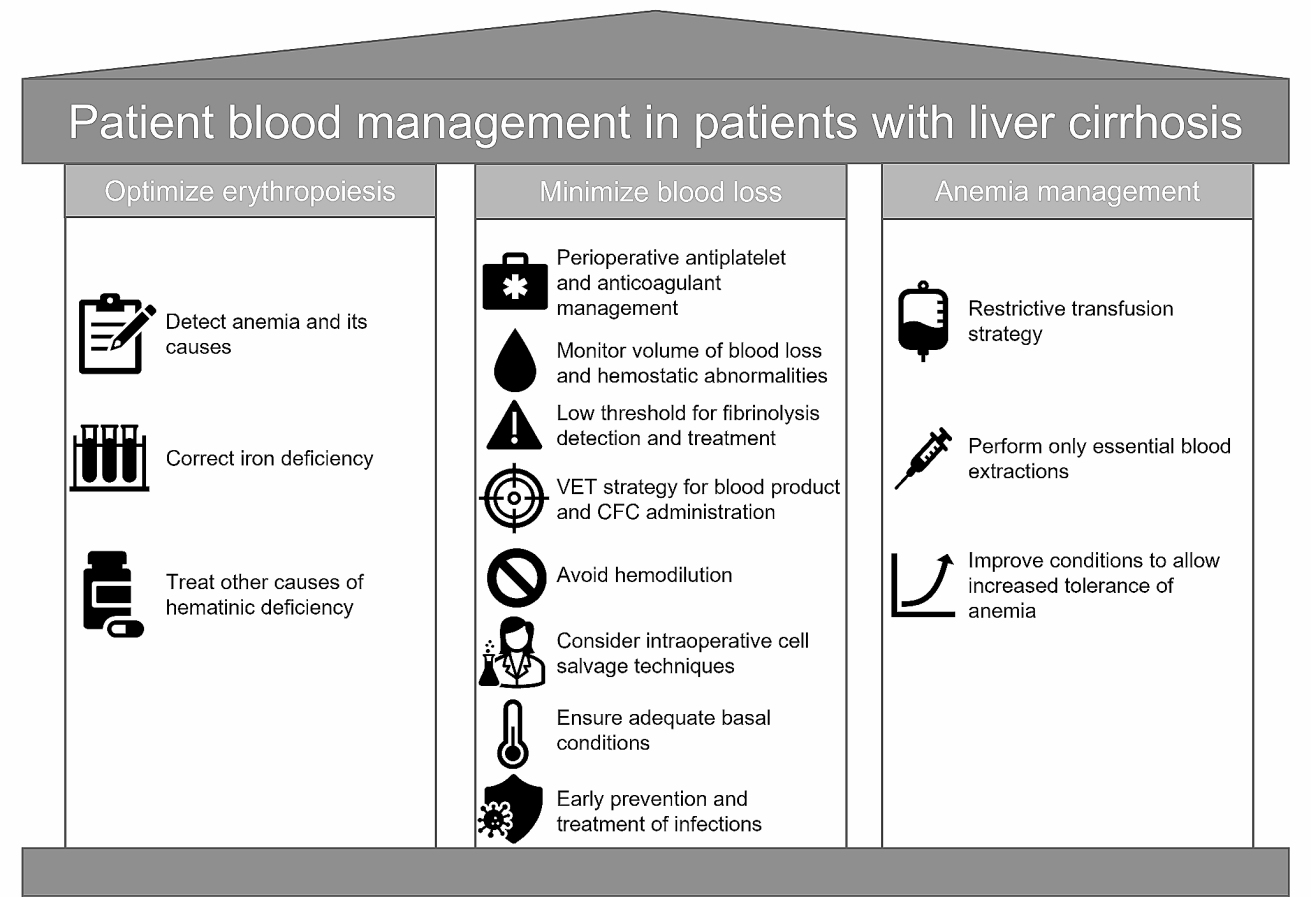
Patient blood management overview CFC, coagulation factor concentrate; VET, viscoelastic testing

In this patient practicum, we aim to provide a narrative review and expert opinion on the typical management of periprocedural and transplant-related bleeding in patients with cirrhosis, based on the principles of patient blood management, which prioritize optimization of erythropoiesis, minimization of bleeding and blood loss, and management of anemia (Fig. 1).

## Management of periprocedural bleeding in patients with cirrhosis

### Management of bleeding in cirrhotic patients undergoing invasive procedures (non-transplant surgeries)

Even when minor procedures are planned, patients with cirrhosis are traditionally perceived as having an increased risk of bleeding complications. Some procedures are considered low-risk, including central line insertion and paracentesis or thoracentesis, whereas others such as liver biopsy, transjugular intrahepatic portosystemic shunt (TIPS) placement, and surgery are considered high-risk procedures for these patients [3]. The risk factors related to the patient include the severity and the degree of compensation of liver disease (e.g., compensated, acute decompensation, acute-on-chronic liver failure), usually associated with the prolongation of SCTs and thrombocytopenia, which are not reliable as direct predictors of procedure-related bleeding. Hepatic decompensation can disturb the fragile hemostatic balance of the cirrhotic patient, moderately increasing the risk of bleeding/thrombotic complications [3]. Besides other factors such as comorbidities (renal failure) or antithrombotic medication, the risk of bleeding during invasive procedures in cirrhotic patients is likely due to portal hypertension, related to the increase in portal pressure [3]. As a preventive step for bleeding-related risks alone, prophylaxis for low-risk procedures (where major bleeding is expected in < 1.5% of cases; significant bleeding can be easily managed) is not usually recommended [3–5]; however, it can be considered in selected cases as directed by the treating physician.

Preprocedural evaluation should consider a patient’s previous bleeding history, inherited bleeding disorders, ongoing illnesses, and antithrombotic therapies, all of which can reveal increased bleeding risk. Modifiable factors (such as antithrombotic drug use, acute kidney injury, infection) should be identified and, if possible, corrected prior to invasive procedures. The severity of the liver disease and estimation of survival with or without TIPS or transplant should be also assessed before invasive procedures, via common scoring systems such as Child-Pugh and model for end-stage liver disease (MELD) to comprehensively assess the rate of disease progression and periprocedural risk [3, 5]. Although not useful to predict the bleeding risk, SCTs and platelet count should be assessed before both low-risk bleeding procedures and invasive procedures, as they offer an image of the baseline patient status for comparison with later tests and can roughly guide the clinician to the first measures of correction in the event of significant procedural-related bleeding [4]. Preprocedural coagulation management of bleeding risk is complex, with no specific values for INR, platelet, or VET thresholds as reliable guidance for increased bleeding risk [6].

Taking into account the well-known risks of allogenic blood product transfusion, with the additional risk of volume overload, exacerbation of portal hypertension, and bleeding risk in cirrhotic patients receiving fresh frozen plasma, preprocedural correction of hemostasis in patients with cirrhosis is generally not recommended; depending on the procedure and on the patient, an individualized approach for correction of platelet counts, fibrinogen levels and/or clot firmness is recommended [3, 4].

Even with its known limitations, VET can be used to aid individual assessments of bleeding risk, allowing a better selection of patients in whom to consider preprocedural prophylaxis and for guiding bleeding management in cirrhotic patients during invasive procedures [2, 7]. For example, in patients with decompensated cirrhosis, thromboelastography performed prior to procedures indicated that maximum amplitude was noticeably reduced in patients who later experienced major bleeding (< 30 mm) compared with those who had little or no bleeding [7]. Employing a VET-guided approach to preprocedural bleeding risk assessments could, therefore, focus usage of blood products and factor concentrates on only patients with the highest risk of bleeding, thus avoiding an unnecessary and risky procedure in patients that may not need it, and improving both overall resource utilization and cost-effectiveness. This has further been demonstrated in a randomized controlled trial of children with cirrhosis who underwent invasive procedures, in which rotational thromboelastometry (ROTEM)-based transfusion strategies reduced the requirement for blood component transfusion and was found to be cost-effective (*p* = 0.002) [8].

Although neither SCT or VET can reliably predict procedural bleeding risk, VET can offer some guidance and has the potential to become a tool useful to predict intra or postprocedural bleeding in the future, if high quality studies are able to provide evidence on specific cut-offs for VET parameters associated with bleeding in cirrhotic patients. Due to the convenience of VET and the short turnaround time, the management of cirrhotic patients undergoing procedures with a high risk of bleeding, such as surgery, should include the use of VET for a guided and targeted therapy of coagulation defects associated with bleeding. In clinical practice hyperfibrinolysis is not detected by SCTs, but can be revealed by VET (though with a low sensitivity for moderate and mild hyperfibrinolysis) [3]. Antifibrinolytics are not routinely recommended for bleeding prophylaxis and should be administered in case of procedural bleeding when hyperfibrinolysis is suspected (based on clinical assessment) or diagnosed by VET [3, 5].

To minimize procedure-related and even spontaneous bleeding risk, a careful fluid management strategy should be adopted, avoiding hypervolemia, which is associated with increased portal pressure and higher bleeding risk. A restrictive red blood cell transfusion policy is also essential, with maintenance of hemoglobin levels of 7─8 g/dL as the current standard of care to support both adequate oxygen delivery and balance between the bleeding tendency and hematocrit levels in most patients [4].

#### Management of bleeding in cirrhotic patients undergoing transplant surgery

Prior to transplantation, patients should be thoroughly assessed for bleeding risk, similar to the preprocedural assessment performed before other invasive procedures described earlier. Blood loss and blood component requirements during liver transplantation can be predicted to a certain extent, based on preoperative assessment of hemoglobin, fibrinogen levels, or clot firmness on VET [9]. There is not enough evidence regarding the cut-off values of various preoperative hemostatic parameters associated with clinically significant intraoperative blood loss; however, preoperative anemia is clearly related to perioperative blood transfusion. Anemia pre-transplantation and in the early period following liver transplant is a common complication in patients with cirrhosis. To prevent the development or aggravation of anemia, blood extractions should be kept to a minimum, with only essential blood testing advised. Tolerance to anemia should be built by optimizing hemodynamics, adequate oxygenation, and the avoidance of situations associated with increased oxygen consumption. Current guidelines recommend that in patients with cirrhosis who are undergoing invasive procedures, hemoglobin levels should be optimized by treating relevant iron, folic acid, and deficiencies in vitamins B6 and B12 to reduce the use of allogenic blood products and their associated complications [4].

**Fig. 2:**
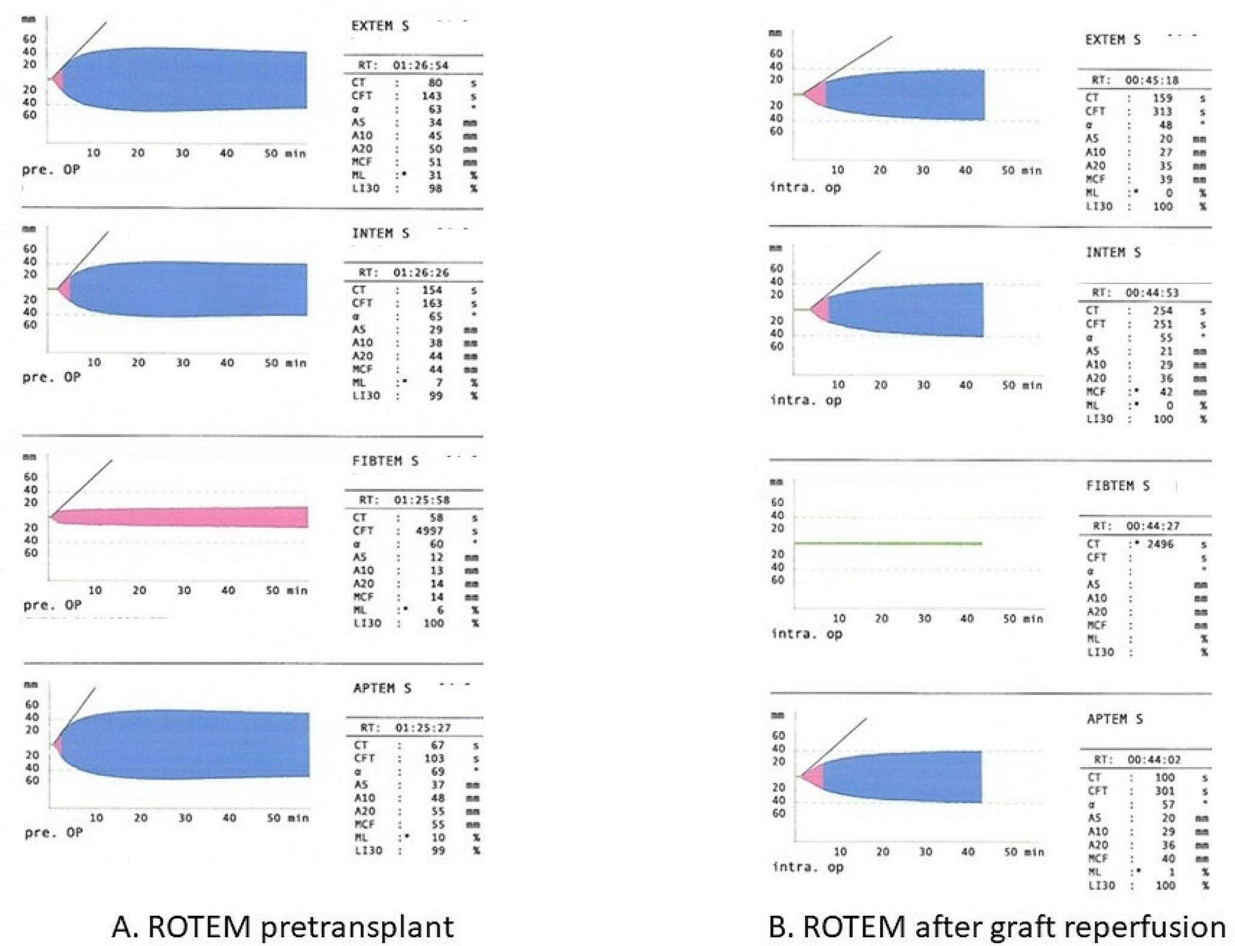
ROTEM tests in patients undergoing liver transplantation for primary biliary cholangitis **A**) pretransplant and **B**) after graft reperfusion ROTEM, rotational thromboelastometry

Hemostasis should be monitored perioperatively to inform bleeding management decisions in liver transplant recipients. If possible, baseline VET results should be recorded, and testing should be repeated shortly after reperfusion and also at surgical wound closure, in case of bleeding to guide administration of blood products and coagulation factor concentrates, and finally after therapeutic interventions for coagulopathy correction [10]. While the administration of antifibrinolytic agents is not routinely recommended as prophylaxis, antifibrinolytic therapy is advised when fibrinolysis is clinically suspected or indicated by VET results [10]. The administration of blood products and factor concentrates for coagulopathy correction should be based on algorithms developed for liver transplant patients, guided by VET if available [10]. In a recent randomized multicenter trial, plasma fibrinogen level and maximum clot firmness (MCF) FIBTEM values were shown to be decreased during liver transplantation, indicating consumption of fibrinogen [11]. The decrease in fibrinogen level and MCF FIBTEM was more pronounced after graft reperfusion and as a result, nearly 70% of patients required fibrinogen replacement at this time [11]. This is demonstrated by the ROTEM tests of patients undergoing liver transplantation in Fig. 2.

Volume overload is associated with increased portal pressure and increased risk of bleeding and should be avoided in liver transplant patients [4]. Careful monitoring and maintenance of adequate volume status and hemodynamic parameters are extremely important not only intraoperatively – to avoid volume overload associated with increased bleeding during pre-anhepatic phase and with graft congestion after reperfusion, or volume depletion associated with vital organ hypoperfusion – but also in the early postoperative period to ensure adequate graft perfusion.

The use of VET, cell salvage, and educational interventions are recommended to reduce the need for blood product transfusion. As with other procedures for patients with cirrhosis, during liver transplantation, a restrictive approach to transfusion, where possible, is the current standard of care to ensure hemoglobin levels of 7─8 g/dL are maintained [10]. In the early post-transplant period hemostasis should be carefully monitored, as the risks for bleeding, thrombotic events, graft dysfunction, or other complications related to altered hemostatic balance are considerable. For this reason, patients should be monitored regularly with SCTs and with VET, if available, and the hemostatic management should be individualized [10].

## Conclusions

Patients with cirrhosis who receive VET-guided bleeding management when undergoing invasive procedures, non-transplant surgeries and liver transplantation experience decreased transfusion of allogenic blood products and improved outcomes. When preparing patients with cirrhosis for invasive procedures, where possible, patients should be assessed for bleeding risk prior to any planned procedures and transfusion strategies should be restrictive. Among other measures included in the patient blood management strategy, preoperative anemia diagnosis and correction should receive particular attention and should be implemented as standard of care. In order to improve outcomes in this fragile patient population, more high-quality studies are urgently needed to bring the evidence necessary to build formal guidelines for anemia diagnosis and correction, and for bleeding management, specifically adapted for patients with cirrhosis.
